# Clinical Effects of Dental Caries on the Quality of Life of Paediatric Patients Aged 8–10 Years: Utilisation of the PUFA Index

**DOI:** 10.3290/j.ohpd.b4009717

**Published:** 2023-04-04

**Authors:** Ghalia Y. Bhadila, Nada J. Farsi, Hala Aljishi, Dua’a Telmisani, Sara M. Bagher

**Affiliations:** a Assistant Professor, Department of Paediatric Dentistry, King Abdulaziz University, Jeddah, Saudi Arabia. Study design, interpreted the data, edited and reviewed the manuscript.; b Associate Professor, Department of Dental Public Health, King Abdulaziz University, Jeddah, Saudi Arabia. Study design, statistical analysis, interpreted the data, critically reviewed the manuscript.; c General Dentist, King Abdulaziz University, Jeddah, Saudi Arabia. Collected the data, wrote the manuscript.; d Associate Professor, Department of Paediatric Dentistry, King Abdulaziz University, Jeddah, Saudi Arabia. Study design, interpreted the data, and critically reviewed the manuscript.

**Keywords:** dental caries, oral health, paediatric dentistry, quality of life, school-age population

## Abstract

**Purpose:**

Saudi children have poor oral health; however, little data are available on the effects of dental caries and its clinical complications on the oral health-related quality of life (OHRQoL) in school-aged children. This study evaluated the impact of caries and its clinical effects on the OHRQoL of a sample of 8- to 10-year-old children attending King Abdulaziz University Hospital.

**Materials and Methods:**

The following variables were assessed for each child: sociodemographic data, OHRQoL using an Arabic-validated Child Perception Questionnaire for 8- to 10-year-old children (CPQ8–10), and two global health rating questions. Caries and its clinical effects on oral health were also assessed using the decayed-missing-filled teeth (dmft/DMFT) and pulpal involvement, ulceration, fistula, and abscess (pufa/PUFA) indices. Descriptive statistics of the sociodemographic variables and responses to the CPQ8–10 questions are presented as absolute values and percentages. The CPQ8–10 scores between children with different dmft/DMFT and pufa/PUFA scores were compared.

**Results:**

In total, 169 children participated in this study. The means ± SD of dmft and DMFT were 5.03 ± 2.5 and 2.35 ± 1.7, respectively. However, the pufa and PUFA scores were 1.03 ± 1.6 and 0.05 ± 0.2, respectively. The most common oral health complaint affecting OHRQoL was food stuck to the teeth. Participants with higher dmft and pufa/PUFA scores had statistically significantly higher CPQ8–10 scores than did their counterparts.

**Conclusion:**

High dmft and pufa/PUFA scores have a statistically signifcantly negative effect on the OHRQoL among healthy 8- to 10-year-old children. Worse global health ratings correlate with lower OHRQoL.

Oral health is an essential part of overall well-being and includes the ability to speak, smile, smell, taste, touch, chew, swallow, and convey a range of emotions through facial expressions with confidence and without pain, discomfort, or disease of the craniofacial complex.^12^ In recent decades, several instruments to measure the influence of oral health conditions on the quality of life have been developed and validated, such as the Oral Impacts on Daily Performance Index,^23^ Scale of Oral Health Outcomes for 5-year-old children,^24^ and Early Childhood Caries Impact Scale (ECOHIS).^14^ In 2002, an age-specific Child Perception Questionnaire (CPQ) was developed for children aged 6–7, 8–10, and 11–14 years (CPQ6–7, CPQ8–10, CPQ11–14, respectively).^15^ The age-specific CPQ8–10 has been utilised in previous studies to evaluate the oral health-related quality of life (OHRQoL) among 8- to 10-year-old children.^2,19^ The English version consists of 25 questions that have been validated in Canada and translated into different languages,^5,6^ including Arabic.^2^ The Arabic version of the CPQ8–10, together with two global health rating questions, was recently validated by Al-Blaihed et al,^2^ and their reliability was tested.

The decayed-missing-filled teeth (dmft/DMFT) index is a rapid and convenient tool used worldwide for dental caries assessment.^10^ However, using this index, the effects of caries other than filling and extraction are not assessed, and this index provides equal weight to caries of varying severities.^22^ Additionally, caries complications, such as infections, are not assessed in the dmft/DMFT index.^18^ Thus, Monse et al^18^ created an exposed pulpal involvement, ulceration, fistula, and abscess (pufa/PUFA) index to measure the clinical effects of untreated caries. The presence of a visible pulp (P/p), ulceration of the oral mucosa due to root fragments (U/u), fistula (F/f), or abscess (A/a) was assessed and evaluated using the pufa/PUFA index. As pufa/PUFA is a new index, only a few studies have evaluated its use in evaluating the effects of untreated caries and its clinical effects on OHRQoL.^3,19,21^

Based on a recent meta-analysis, in Saudi Arabia, approximately 84% and 72% of children aged 5–7 and 12–15 years, respectively, were diagnosed with dental caries.^1^ Despite the high caries prevalence among children in Saudi Arabia, few studies have investigated the clinical effects of caries on their OHRQoL. Thus, this study aimed to assess the impact of caries and its clinical effects using both dmft/DMFT and pufa/PUFA indices on the OHRQoL of a sample of 8- to 10-year-old children attending King Abdulaziz University Hospital. We hypothesised that dental caries and its clinical effects using dmft/DMFT and pufa/PUFA indices are associated with lower OHRQoL among a sample of 8- to 10-year-old children. The null hypothesis is that dental caries and its clinical effects using dmft/DMFT and pufa/PUFA indices are not associated with OHRQoL among a sample of 8- to 10-year-old children.

## Materials and Methods

This cross-sectional study was conducted in accordance with the Strengthening the Reporting of Observational Studies in Epidemiology recommendations.^25^ This study was approved by the Research Ethics Committee at King Abdulaziz University Hospital (approval no. 006-01-22). A consecutive sample of all parents of healthy, 8- to 10-year-old children who visited King Abdulaziz University Hospital between January and April 2022 were invited to participate in this study. King Abdulaziz University Hospital is the largest governmental dental educational institute in Jeddah city, which is considered the second largest city in the country.^17^ Before enrolling any child in this study, a cover letter and consent form were provided to the parents of eligible children, clarifying the rationale and steps of this study. Only children of consenting parents were included in this study. Oral assent was obtained from each participating child. For children to be included, they had to be healthy and Arabic had to be their native language and that of their parents. Children who were unwilling to participate in this study or those receiving orthodontic treatment were excluded from this study.

The sample size was determined using G-power software (https://www.psychologie.hhu.de/arbeitsgruppen/allgemeine-psychologie-und-arbeitspsychologie/gpower).^11^ The calculation was based on a significance level of 0.05, aiming at a statistical power level of 80% and assuming an effect size of 0.25. The outcome was the CPQ8–10 score, which is a continuous variable, being compared between groups of children with varying dmft/DMFT and pufa/PUFA scores. Based on this information, approximately 159 participating children and their parents were required, and 10 participants were added to accommodate for missing values and nonresponses. Thus, the total sample size was 169 participants.

### Measured Variables

The parents and participating children were interviewed by a single trained general dentist (H.A). The interviews were conducted in two parts. In the first part, the following sociodemographic data of the participating children were collected from one of the parents: age, gender, type of school, nationality, parental educational level, average household monthly income, and house ownership. In addition, the frequency of children’s toothbrushing was recorded. The second part included two Arabic-validated global health rating questions and the CPQ8–10 questionnaire^.2^ The two global health rating questions were answered by the participating parents to assess a. the parental perception of the child’s oral health and b. the effects of their child’s oral health on their overall well-being, with the response options being 0 = excellent, 1 = very good, 2 = good, 3 = acceptable, 4 = poor, and 0 = not at all, 1 = very little, 2 = somewhat, 3 = a lot, and 4 = very much, respectively.^9^

The Arabic-validated CPQ8–10 questionnaire was administered by interviewing the participating children, and parents were instructed not to assist their children or interfere with the interview process. The CPQ8–10 comprised 25 questions distributed across four domains: (i) oral symptoms, (ii) functional limitations, (iii) emotional well-being and (iv) social well-being. The first three sections included five questions each, and the last section included 10 questions. Each of the 25 questions was scored based on the frequency on a five-point Likert scale: 0 = never, 1 = once or twice, 2 = sometimes, 3 = often, and 4 = every day or almost every day.

Clinical examination to assess caries was performed using the dmft/DMFT index by a single calibrated and trained general dentist (DT), who was trained by a paediatric dentist (GB). Training and calibration were performed by examining 10 randomly selected 8- to 10-year-old children. The same patients were re-examined after 2 weeks, and intraclass correlation coefficients were estimated to be 100% for dmft/DMFT and pufa/PUFA.

Oral examination was completed under clinical light with the aid of a mouth mirror, blunt community periodontal index probe (Shepherd’s Hook probe, Nordent; Elk Grove Village, IL, USA) and dental gauze to dry the teeth. A dental examination was performed while the child was lying on the dental chair, and the following were recorded: (i) dmft/DMFT, which was used for the detection of teeth that were decayed, missing, or permanently filled teeth due to caries, and (ii) pufa/PUFA to evaluate the clinical effects of tooth decay. The pufa/PUFA index was recorded to detect pulpally involved teeth, ulcers due to sharp parts of carious teeth, fistulas linked to badly decayed teeth, and abscesses related to carious teeth.^18^

### Statistical Analysis

Descriptive statistics of sociodemographic variables and responses to the CPQ8–10 questions are presented as counts (n) and percentages, respectively. For continuous variables, median, mean and standard deviation (SD) were computed. The CPQ8–10 score was calculated by summing the responses to different questions. For each question, the responses never, once/twice, sometimes, often, and every day were coded as 0, 1, 2, 3, and 4, respectively. The scores ranged from 0 to 100, with 100 indicating the worst QoL. The normality of the CPQ8–10 scores was assessed by visually inspecting a histogram and a Q-Q plot, as well as by interpreting the results of skewness and kurtosis tests, which all indicated that the scores had a non-normal distribution. Thus, nonparametric tests were used in this study. CPQ8–10 scores among children with different dmft/DMFT scores (0, 1–5, ≥ 6) were compared using the Kruskal-Wallis test. When results were statistically significant, Dunn’s test of multiple comparisons was performed using rank sums. The CPQ8–10 scores of children with varying pufa/PUFA scores (0 ≥ 1) were also compared using the Wilcoxon rank-sum test. The means ± SD, medians, and interquartile ranges (IQRs) were calculated separately for the participating children according to their parental responses to the global health rating question “How would you describe the health status of your child’s teeth, lips, mouth, and jaws in general?” Spearman’s correlation coefficient was also calculated to assess the correlation between the responses to the global health questions and CPQ8–10 scores. There were no missing data. Statistical significance was set at p ≤ 0.05. Stata version 12.1 (Stata; College Station, TX, USA) was employed to perform statistical analyses.

## Results

The data supporting the findings of this study, as well as the Arabic-validated CPQ8–10 questionnaire, are available from the corresponding author upon request.

This study included 169 children aged 8–10 years. Table 1 shows the sociodemographic characteristics of the participating children and their parental responses to the two global health rating questions. Fifty-three percent of the participating parents reported that their children had excellent or very good oral health. Only 2% of the participating parents considered that oral health does not affect the child’s overall well-being, and 9% reported that it has a very little effect.

**Table 1:** Sociodemographic characteristics and responses to global health rating questions of the participants (n = 169)

Variables		n (%)
Age in years	8	62 (36.7)
	9	51 (30.2)
	10	56 (33.1)
Gender	Male	84 (49.7)
	Female	85 (50.3)
Type of school	Private	38 (22.5
	Public	131 (77.5)
Nationality	Saudi	127 (75.2)
	Non-Saudi	42 (24.9)
Mother’s occupation	Employed	64 (37.9)
	Unemployed	105 (62.1)
	Retired	0 (0.0
Father’s occupation	Employed	150 (88.8)
	Unemployed	10 (5.9)
	Retired	9 (5.3)
Average household monthly income (SAR)	< 4000	44 (26.0)
	≥4000	125 (73.7)
House ownership	No	104 (61.5)
	Yes	65 (38.5)
Daily brushing frequency	None	4 (2.4)
	Once	72 (42.6)
	Twice or more	93 (55.0)
How would you describe the health status of your child’s teeth, lips, mouth, and jaws in general?	Excellent	50 (29.6)
	Very good	40 (23.7)
	Good	8 (4.7)
	Acceptable	38 (22.5)
	Poor	33 (19.5)
In general, how does the health of your mouth and teeth affect your child’s overall well-being?	Not at all	3 (1.8)
	Very little	15 (8.9)
	Somewhat	30 (17.8)
	A lot	26 (15.4)
	Very much	95 (56.2)

SAR: Saudi Riyal.

The mean, SD, median, and IQR of the dmft, DMFT, pufa, and PUFA indices are presented in Table 2. Table 3 illustrates the participating children’s responses to the CPQ8–10 questions. The CPQ8–10 scores among the participating children ranged from 0 to 48 out of 100, with a mean score of 15.5 ± 10.3. The most common oral health complaint affecting their OHRQoL was food stuck to their teeth (63%), followed by bad breath in their mouth (38.5%). None of the participating children reported that their oral health problems affected school (completing homework, lack of desire for reading or speaking in class, difficulty paying attention in school).

**Table 2:** Mean ± SD and medians of dmft, DMFT, pufa and PUFA indices of the participants (n= 169)

Variables	Primary teeth		Permanent teeth	
	Mean ± SD	Median (IQR)	Mean ± SD	Median (IQR)
d/D	1.95 ± 2.0	2 (0–3)	0.92 ± 1.2	0 (0–2)
m/M	1.23 ± 1.5	1 (0–2)	0	0 (0–0)
f/F	1.85 ± 1.9	1 (0–3)	1.44 ± 1.6	1 (0–3)
dmft/DMFT	5.03 ± 2.5	5 (3–7)	2.35 ± 1.7	2 (1–4)
p/P	0.99 ± 1.6	0 (0–2)	0.05 ± 0.2	0 (0–0)
u/U	0.0	0 (0–0)	0.0	0 (0–0)
f/F	0.02 ± 0.2	0 (0–0)	0.0	0 (0–0)
a/A	0.01 ± 0.1	0 (0–0)	0.0	0 (0–0)
pufa/PUFA	1.03 ± 1.6	0 (0–2)	0.05 ± 0.2	0 (0–0)

d/D: decayed teeth; m/M: missing teeth; f/F: filled teeth. p/P: pulpally involved teeth; u/U: ulcers; f/F: fistula; a/A: abscess.

**Table 3:** The participating children’s response to quality-of-life questionnaire CPQ 8-10

Questions	Never n (%)	Once/twice n (%)	Sometimes n (%)	Often n (%)	Always n (%)
1. Pain in your teeth or mouth?	53 (31.4)	19 (11.2)	44 (26.0)	3 (1.8)	50 (29.6)
2. Sore areas (ulcers) in your mouth?	149 (88.2)	12 (7.1)	6 (3.6)	0 (0.0)	2 (1.2)
3. Do you have pain in your teeth when you drink cold drinks or when you eat hot food?	68 (40.2)	15 (8.9)	39 (23.1)	2 (1.2)	45 (26.6)
4. Does food stick to your teeth?	25 (14.8)	0 (0.0)	33 (19.5)	5 (3.0)	106 (62.7)
5. Do you have bad breath in the mouth?	60 (35.5)	11 (6.5)	29 (17.2)	4 (2.4)	65 (38.5)
6. Do you take longer than others to eat your meal because of your teeth or mouth?	147 (87.0)	4 (2.4)	14 (8.3)	0 (0.0)	4 (2.4)
7. Do you have difficulty chewing or cutting food such as apples, corn and meat because of your teeth or mouth?	136 (80.5)	7 (4.1)	15 (8.9)	3 (1.8)	8 (4.8)
8. Do you have difficulty eating the foods you love because of your mouth or teeth?	149 (88.2)	4 (2.4)	13 (7.7)	0 (0.0)	3 (1.8)
9. Do you have difficulty pronouncing some words because of your teeth or mouth?	158 (93.5)	1 (0.6)	5 (3.0)	0 (0.0)	5 (3.0)
10. Do you have difficulty sleeping at night because of your teeth or mouth?	99 (58.6)	16 (9.5)	31 (18.3)	2 (1.2)	21 (12.4)
11. Are you bothered by your teeth or mouth?	112 (66.3)	8 (4.7)	25 (14.8)	0 (0.0)	24 (14.2)
12. Do you feel frustrated because of your teeth or your mouth?	124 (73.4)	1 (0.6)	20 (11.8)	0 (0.0)	24 (14.2)
13. Are you ashamed because of your teeth or your mouth?	130 (76.9)	4 (2.4)	23 (13.6)	1 (0.6)	11 (6.51)
14. Are you interested in what other people think of your teeth or mouth?	135 (79.9)	6 (3.6)	21 (12.4)	2 (1.2)	5 (2.96)
15. Are you worried that you don’t look as good as others because of your teeth or mouth?	140 (82.8)	5 (3.0)	17 (10.1)	1 (0.6)	6 (3.6)
16. Do you miss school because of your teeth or your mouth?	148 (87.6)	17 (10.1)	4 (2.4)	0 (0.0)	0 (0.0)
17. Do you have difficulty doing your homework because of your teeth or mouth?	169 (100.0)	0 (0.0)	0 (0.0)	0 (0.0)	0 (0.0)
18. Do you have difficulty paying attention at school because of your teeth or mouth?	169 (100.0)	0 (0.0)	0 (0.0)	0 (0.0)	0 (0.0)
19. Do you have no desire to speak or read aloud in class because of your teeth or your mouth?	169 (100.0)	0 (0.0)	0 (0.0)	0 (0.0)	0 (0.0)
20. Do you have you try not to smile or laugh when you are with other children because of your teeth or mouth?	144 (85.2)	9 (5.3)	14 (8.3)	1 (0.6)	1 (0.6)
21. Do you have no desire to talk to other children because of your teeth or your mouth?	157 (92.9)	1 (0.6)	10 (5.9)	0 (0.0)	1 (0.6)
22. Do you have not want to be around other kids because of your teeth or your mouth?	157 (92.9)	4 (2.4)	7 (4.1)	0 (0.0)	1 (0.6)
23. Do you have you avoid sports activities and clubs because of your teeth or mouth?	165 (97.6)	2 (1.2)	1 (0.6)	0 (0.0)	1 (0.6)
24. Do you children tease you or call you names because of your teeth or mouth?	149 (88.2)	2 (1.2)	15 (8.9)	0 (0.0)	3 (1.8)
25. Do you children ask you questions about your teeth or your mouth?	129 (76.3)	9 (5.3)	22 (13.0)	1 (0.6)	8 (4.7)

Table 4 shows the CPQ8–10 scores for the different dmft, DMFT, pufa, and PUFA index categories and the correlation between the CPQ8–10 scores and dmft, DMFT, pufa, and PUFA scores. Participants with higher dmft score had significantly higher CPQ8–10 mean scores than those with lower dmft score. The difference in the mean scores between participating children with a dmft score of 1–5 and a dmft score ≥ 6 was statistically significant (p < 0.001). Children with pufa/PUFA score ≥ 1 had significantly higher CPQ8–10 mean scores those with a score of zero pufa/PUFA (pufa, p < 0.001; PUFA, p = 0.042).

**Table 4:** The correlation between the participating children’s CPQ8–10 mean ± SD, median scores, and dmft/DMFT and pufa/PUFA indices

Index score		n	CPQ8–10 score		p-value	r^†^	p-value
			Mean (SD)	Median (IQR)			
dmft	0	9	7.67 (3.6)^a^	7^a^ (6–8)	<0.001#	0.371	<0.001*
	1–5	83	13.02 (8.6)^a^	10^a^ (7–18)			
	≥ 6	77	19.14 (11.3)^b^	16^b^ (12–26)			
DMFT	0	32	13.19 (8.3)	11 (8–17)	0.329	0.136	0.078
	1–5	134	16.22 (10.8)	14 (8–24)			
	≥ 6	3	9.67 (1.5)	10 (8–11)			
pufa	0	100	13.08 (8.7)	11 (7.5–16)	<0.001^	0.305	<0.001*
	≥1	69	19.07 (11.5)	18 (10–26)			
PUFA	0	162	15.2 (10.2)	12 (8–21)	0.0419^	0.157	0.042*
	≥1	7	23.1 (11.7)	18 (14–38)			

CPQ: Child Perceptions Questionnaire. IQR: interquartile range. #Kruskal-Wallis test. ^Wilcoxon rank-sum test. Dunn’s test of multiple comparisons using rank sums: similar letters indicate no statistically significant difference. Different superscript letters indicate statistically significant difference. ^†^Spearman’s correlation coefficient. *Statistically significant at p<0.05.

Table 5 shows the correlation between the CPQ8–10 scores and global health rating question responses. Parents who perceived the oral health condition of their child to be excellent had a median CPQ8–10 score of 9, which increased to 14 and 20.5 for those reporting very good and good, respectively. Global health ratings were correlated with CPQ8–10 scores; worse global ratings were correlated with higher CPQ8–10 scores (r = 0.216, p = 0.0048).

**Table 5:** The correlation between the participating children’s CPQ8-10 mean ± SD, median scores, and global health rating question responses (parents’ answers)

	n	Mean (SD)	Median (IQR)	P-value^	r^†^	p-value
Global health rating question “How would you describe the health status of your child’s teeth, lips, mouth, and jaws in general?”						
Excellent	50	12.3 (9.2)	9 (6–16)^a^	0.024	0.216	0.0048*
Very good	40	15.6 (9.3)	14 (8–21)^a^			
Good	8	22.5 (13.5)	20.5 (10–36.5)^a^			
Acceptable	38	16.1 (9.7)	15 (10–23)^a^			
Poor	33	18.0 (11.8)	16 (9–24)^b^			
How much does the condition of your child’s teeth, lips, jaws or mouth affect his overall health?						
Not at all	3	17 (12.1)	15 (6–30)		-0.038	0.624
Very little	15	16.1 (9.4)	14 (10–20)			
Somewhat	30	14.8 (7.7)	13.5 (9–18)			
A lot	26	15.5 (11.8)	12 (6–24)			
Very much	95	15.6 (10.9)	13 (8–22)			

CPQ: Child Perceptions Questionnaire; IQR: interquartile range. ^†^Spearman’s correlation coefficient. ^Kruskal-Wallis test. Dunn’s test of multiple comparisons using rank sums: different superscript letters indicate statistically significant differences. *Statistically significant at p<0.05.

## Discussion

This cross-sectional study assessed the effects of caries and its clinical effects using both dmft/DMFT and pufa/PUFA indices on the OHRQoL of a sample of children aged 8–10 years attending King Abdul Aziz University Hospital. The findings of the study reject the null hypothesis, as there was a statistically significantly higher CPQ8–10 mean score, indicating worse OHRQoL, for children with higher dmft and pufa/PUFA scores than for children with lower dmft and pufa/PUFA scores.

In this study, the mean dmft and DMFT scores were 5.03 and 2.35, respectively. These findings are in agreement with a systematic review that reported dmft and DMFT scores of 5.38 and 3.34, respectively, among a Saudi population.^16^ However, no other studies have reported the pufa/PUFA mean scores for this age group in Saudi Arabia. For permanent teeth, the mean DMFT (2.35 ± 1.7) and PUFA scores (0.05 ± 0.2) were found to be lower than those of the primary teeth dmft (5.03 ± 2.5) and pufa (1.03 ± 1.6), which indicates the worse clinical condition of primary dentition among the participants. This can be attributed to the age group included. Children were at a mixed dentition stage, with the permanent teeth still unerupted or erupted but had not been exposed to cariogenic challenges in the oral cavity for any appreciable length of time.

In this study, participants with a dmft score ≥ 6 reported the statistically significantly highest CPQ8–10 mean scores, indicating worse OHRQoL among the sample. Although not statistically significant, participants with a DMFT score ≥ 6 reported lower CPQ8–10 mean score compared to those with a DMFT score between 1 and 5. Nevertheless, this should be interpreted with caution because the number of participants with DMFT score ≥ 6 was only three, whereas the number of participants with DMFT score between 1–5 was 134.

Data not provided by the dmft/DMFT were provided using pufa/PUFA, that is, the clinical effects of caries that require urgent dental treatment. In the current study, a pufa/PUFA index of ≥ 1 was statistically significantly associated with lower OHRQoL among the participating children. This is consistent with the results of a previous study which assessed the impact of untreated caries and its clinical effects on OHRQoL using the CPQ8–10 among Brazilian school-aged children, where caries had a significant effect on the oral symptoms and functional limitation domains of the CPQ8–10.^19^ This also agrees with the result of a study conducted in India that investigated the impact of early childhood caries and its clinical effects using the pufa index on the OHRQoL of infants and toddlers using the ECOHIS.^21^ Those authors reported that a higher pufa score negatively affected the OHRQoL of infants and toddlers.^21^ However, the findings in the present study were not in agreement with those of a study conducted among Saudi children aged 11–14 years, where no association between PUFA score and OHRQoL was found.^3^

Quality-of-life–related studies in children provide insight and a better understanding of the effects of oral health on the everyday activities of children.^13^ Based on a review by Mulla^20^ published in 2021, which reviewed the effects of different oral diseases and conditions on the OHRQoL among populations in Saudi Arabia, most of the published studies focused on older age groups, and none of the available studies evaluated the impact of caries and its clinical effects on the OHRQoL among healthy young children.^20^ In 2019, Al-Zahrani et al^4^ conducted a study on healthy 12- to 15-year-old children in Saudi Arabia to investigate the effects of caries (using the DMFT score), gingival inflammation, and plaque accumulation on OHRQoL. In that study, the mean DMFT score was 0.49 ± 0.61, and approximately 75% of the participants reported that their oral health had affected their daily performance within the last 3 months.^4^ Eating and enjoying food were among the most frequent daily activities affected by oral health status (54.4%).^4^ This is similar to our findings, in which the most common oral health complaint affecting OHRQoL was food stuck to the teeth. In the current study, the mean DMFT score was 2.35 ± 1.7, which is statistically significantly higher than that reported by Al-Zahrani et al.^4^ This might be due to the differences in the participants’ age groups between the two studies. In the current study, the participants were of both sexes and were recruited from patients who were attending a governmental hospital to receive dental treatment; in the study conducted by Al-Zahrani et al,^4^ the subjects were males only and were recruited randomly from three different schools, which might explain the variability of the findings.

Despite the difference in the participants’ age group, recruitment sites, and cities in Saudi Arabia, the association between higher scores of caries and lower OHRQoL presented in the current study is consistent with those of previous studies.^3,7^ These studies used the Arabic version of the CPQ10–14 and DMFT scores to assess the impact of caries on OHRQoL among school children.^3,7^

Half of the participating parents reported that oral health affected their children’s overall well-being. This finding is consistent with the results of previous studies, which found a statistically positive correlation between the CPQ11–14 scale score and overall well-being in older age groups^7,9^ and in a group of children of similar age groups.^2,6,8^ Similar to Al-Blaihed et al^2^ and Bhayat et al,^7^ we found a statistically significant correlation between worse global health ratings and higher CPQ8–10 scale scores. This suggests that those who responded with “poor” have a statistically significantly higher CPQ8–10 score than those who who rated global health as better than “poor”.

This study has some limitations, for instance, some of the interviewed children reported not clearly understanding certain words, such as “ulcer”. Therefore, the phrase “sore spots” was included next to the word “ulcer” to help them understand its meaning. Furthermore, the questionnaire is still considered lengthy, which makes it inconvenient for some participants. Future studies should evaluate the validity of a shortened version of the original full-length questionnaire, similar to what was done in the CPQ11–14.^7^ Another limitation is that some of the participants could not recall the reason for extraction, whether due to caries, trauma, or other reasons. This might have introduced a recall bias. Also, the DMFT scoring system was used to assess caries without dental radiographs. This might have led to overlooking some interproximal lesions. Additionally, generalisability of these findings could be limited as it is a single-center study. Future studies should consider multicenter data collection. Finally, the association between each domain and the dmft/DMFT and pufa/PUFA scores was not investigated in the current study.

## Conclusion

The findings of this study provide information on the impact of caries and its clinical effects using pufa/PUFA and dmft/DMFT indices on the OHRQoL of 8- to 10-year-old children. High dmft and pufa/PUFA scores had a statistically significantly negative effect on the OHRQoL among healthy 8- to 10-year-old children. Worse global health ratings correlated with lower OHRQoL. Future studies should assess the OHRQoL before and after dental treatment to ensure that the treatment outcomes have improved the oral health status and OHRQoL of patients.
